# Factors associated with past 30-day abstinence from cigarette smoking in a non-probabilistic sample of 15,456 adult established current smokers in the United States who used JUUL vapor products for three months

**DOI:** 10.1186/s12954-019-0293-7

**Published:** 2019-03-25

**Authors:** Christopher Russell, Farhana Haseen, Neil McKeganey

**Affiliations:** Centre for Substance Use Research, 4.04 West of Scotland Science Park, 2317 Maryhill Road, Glasgow, G20 0SP UK

**Keywords:** JUUL, E-cigarettes, Vapor, Quitting, Smoking, Cigarettes, Tobacco harm reduction

## Abstract

**Background:**

JUUL is the fastest growing and highest selling brand of e-cigarette/vapor products in the USA. Assessing the effect of JUUL vapor products on adult smokers’ use of conventional tobacco cigarettes can help inform the potential population health impact of these products.

**Methods:**

Online surveys assessed past 30-day use of conventional cigarettes, JUUL vapor products, and other e-cigarettes/vapor products, monthly for 3 months, in a non-probabilistic sample of 15,456 US adults (21+ years). Participants were established current smokers of conventional cigarettes and recruited at their first purchase of a JUUL Starter Kit in a retail store or through JUUL’s website. Logistic regression models examined factors associated with participants’ odds of reporting past 30-day abstinence from cigarette smoking at the 3-months assessment.

**Results:**

Past 30-day smoking abstinence at the 3-months assessment was reported by 28.3% of the intent-to-treat (ITT) sample (*n* = 15,456) and 47.1% of an efficacy subset sample that completed the 3-months assessment (*n* = 9272). Covariate-adjusted odds for reporting past 30-day smoking abstinence at the 3-months assessment were significantly higher among participants who primarily used Mint or Mango flavored JUULpods (versus Virginia Tobacco flavor) in the past 30 days; exclusively used JUULpods in characterizing flavors (versus tobacco flavors) in the past 30 days; used a JUUL vaporizer on all 30 of the past 30 days; purchased their first JUUL vaporizer in a retail store (versus online); and first purchased a JUUL Starter Kit to help quit smoking completely. Odds for reporting past 30-day smoking abstinence were significantly lower among participants who, at study enrolment, had smoked regularly for ≥ 20 years, smoked ≥ 20 cigarettes per day, and smoked on all 30 of the previous 30 days.

**Conclusions:**

At least 28.3% of adult smokers had quit smoking cigarettes completely after using a JUUL vaporizer for 3 months. More frequent use of a JUUL vaporizer and primary use of JUULpods containing characterizing flavors, particularly Mint and Mango, appears to be important to new JUUL users’ chances of quitting smoking. The impact of banning retail sales of flavored JUULpods on adult smokers’ likelihood of quitting should be closely assessed.

## Background

Tobacco harm reduction (THR) products and policies aim to prevent or reduce harm by promoting substitution of combustible tobacco with less hazardous non-combustible sources of nicotine to smokers who are unable or unwilling to quit smoking in response to conventional tobacco control measures [1]. Tobacco and nicotine products that present a reduced risk of ill health to an individual relative to smoking cigarettes have potential to benefit the health of the whole population to the extent that (i) they are used in place of more harmful tobacco products (e.g., cigarettes) by individuals who currently use such products *and* were unlikely to have quit or reduced their use of such products in the absence of the reduced-risk product, *and* to the extent that (ii) they are not used by individuals who are not current users of more harmful tobacco products *and* would likely have not initiated or re-initiated use of such products in the absence of the reduced-risk product.

Electronic cigarettes (e-cigarettes)—hand-held devices that use battery power to heat a solution of propylene glycol, glycerol, and often flavorings and nicotine, to produce an aerosol that the user inhales—have emerged in the past decade with the greatest potential for meeting criteria for definition as tobacco harm reduction products. Since their introduction to the US market in 2007, e-cigarettes have rapidly grown in popularity among adults in several countries as an alternative to smoking conventional cigarettes [2–6], and the increasing use of e-cigarettes has been associated with significant increases in rates of smoking cessation at the population level [7–10]. E-cigarettes are now the most popular assisted method of quitting smoking in the USA, used in 35% of smokers’ most recent quit attempts [11]. By comparison, nicotine patches or gums were used in 25% of most recent quit attempts. Though data on the safety of long-term use of nicotine by inhalation will not be available until e-cigarettes have been in widespread use for several decades, several US and global health authorities agree that the currently available evidence suggests e-cigarette use is likely to be less harmful than smoking cigarettes. The magnitude of potential reduced harm to the individual user and the potential impact of e-cigarettes on the health of whole populations, however, continues to be debated [12].

JUUL Labs Inc. is a San Francisco-based company that manufactures pre-filled e-liquid pods known as “JUULpods” for use in an electronic nicotine delivery system (ENDS) known as a “JUUL vaporizer.” JUUL is the fastest growing and highest selling e-cigarette/vapor product in the US market, which is sized at approximately $5.5 billion for 2018 [13]. According to Neilsen data, past 52-week retail sales of JUUL products in the US increased from $150.0 million in July 2017 (+ 652.9% versus July 2016) [14] to $1.3 billion in August 2018 (+ 761.4% versus August 2017) [15], making JUUL the first e-cigarette brand to record over $1 billion in sales in a 52-week period through tracked channels. With a past 52-week sales total more than three times higher than its nearest competitor (an e-cigarette called VUSE, $404.0 million), JUUL now has a greater past 52-week share of the US e-cigarette market than all other e-cigarette brands combined, having increased its market share from 17.7% in July 2017 to 55.7% in August 2018 [5]. Neilsen additionally notes these are likely to be underestimates of JUUL’s true sales and market share, as Nielsen does not track sales through several channels where JUUL products are sold, such as online and vape shops. Assessing the potential population health impact associated with the rapid and substantial increase in sales of JUUL vapor products in the USA has become vitally important.

According to JUUL’s website, JUUL vapor products are intended for adult smokers who want to switch from combustible cigarettes. Under the Modified Risk Tobacco Product (MRTP) provision and the drug provisions in section 911 and section 201(g) of the Federal Food, Drug & Cosmetic Act (FD&C) respectively, manufacturers are prohibited from marketing a new tobacco product, including JUUL vapor products, as a safer, healthier, or less risky alternative to smoking tobacco, or effective as an aid to smoking cessation without FDA authorization to make such claims. Yet, anecdotal user testimonies, many of which are shared daily on social media platforms and internet discussion forums dedicated to vaping (e.g., E-Cigarette Forum), suggests many adult smokers in the USA started using a JUUL vaporizer with an intention to use it as an alternative to continuing to smoke regular cigarettes, and many report that, whether intended or not at the outset of use, using a JUUL vaporizer has helped them to quit smoking completely or to cut down the number of cigarettes they smoke. There are no published data, however, on the likelihood that adult tobacco smokers who begin using a JUUL vaporizer then switch completely to use of a JUUL vaporizer, or the likelihood that adult tobacco smokers who begin using a JUUL vaporizer then continue to use a JUUL vaporizer in addition to continuing to smoke conventional cigarettes. Additionally, no data are available on the user characteristics and product use factors that are positively and negatively associated with smokers’ likelihood of quitting smoking through use of a JUUL vaporizer. Previous research has, for example, identified the frequency with which smokers use e-cigarettes and the use of e-cigarettes containing non-tobacco flavors as important determinants of a smokers’ likelihood of completely substituting e-cigarettes for conventional cigarettes [16–21].

Understanding the role that flavors play in the population’s use of e-cigarettes, and the impact that flavored e-cigarette products have on the population’s use of more harmful tobacco products, like conventional cigarettes, has been identified by the US Food and Drug Administration (FDA) as a public health research priority. The ability to inhale e-cigarette vapor aerosol in a vast and growing variety of characterizing flavors—a distinguishable taste or aroma, other than the taste or aroma of tobacco—is thought to be a major feature accounting for the appeal of e-cigarettes to adult smokers as an alternative to continuing to smoke cigarettes. However, the same concerns that led the US Congress to ban the sale of cigarettes with characterizing flavors in 2009 now exist for e-cigarettes. In particular, concerns have been raised, and some evidence has been reported, that non-tobacco flavored ENDS products, particularly fruit and sweet e-liquid flavors, are driving the appeal of e-cigarettes to youth, and that youth who initiate nicotine use through ENDS products will be more likely to subsequently try using more harmful tobacco products that deliver nicotine more efficiently, such as cigarettes [22–28]. FDA Commissioner, Scott Gottlieb, has summarized the need to weigh the potential risks and benefits of flavored ENDS products to the whole population: “On this issue, we see two sides—on the one hand, we need to know the role that flavors, including menthol, play in attracting youth to initiate tobacco use. But on the other hand, we also need to know whether…certain flavors may help adult cigarette smokers switch to potentially less harmful forms of nicotine delivery; for example, when flavors are used in non-combustible products such as electronic nicotine delivery systems. It is possible for flavors to do both harm and good, perhaps in different product types” [29]. Collecting data that characterize the association between adults’ use of flavored JUUL vapor products—the most widely used brand of vapor products in the USA—and their likelihood of quitting smoking in the short and long-term is therefore vitally important to estimating the potential population health impact of these products.

Through six monthly online surveys of a panel of US adult established current smokers recruited at the point of first purchase of a JUUL vaporizer in a retail store or through JUUL’s e-commerce store, this study examined demographic, smoking-related, and JUUL-related factors associated with self-reported past 30-day abstinence from cigarette smoking after ad libitum use of a JUUL vaporizer for three and six months. Of specific interest was the extent to which smokers’ odds of reporting past 30-day abstinence from smoking varied as a function of their frequency and volume of use of JUULpods in six non-tobacco flavors (versus two tobacco flavors). At the point of writing, data collection from the sixth and final monthly survey had not yet completed. Results are therefore reported for the first 3 months of this study.

## Methods

### Sample and recruitment

Eligible individuals were US adults aged 21 years and older who had smoked at least 100 cigarettes in their lifetime, now smoke cigarettes “every day” or on “some days,” and had purchased their first JUUL Starter Kit from a US retail store or through JUUL Labs Inc.’s e-commerce store at http://www.juul.com within the past 7 days. *Veratad Technologies*’ age verification software, *AgeMatch*^*SM*^, was employed by JUUL Labs Inc. to verify, at the point of an attempted online purchase, that individuals were of aged 21 years or older. A JUUL Starter Kit contains a JUUL vaporizer, a USB charging dock, and one e-liquid pod in each of four flavors (1x Virginia Tobacco, 1x Cool Mint, 1x Mango, and 1x Crème Brulee). Each JUULpod contains 5% nicotine by weight, and each pod contains 0.7 ml, equivalent to 59 mg/ml nicotine per pod.

Individuals were invited to participate in this study in two ways. First, JUUL Labs Inc. sent email invitations to 37,536 age-verified adults who had purchased a JUUL Starter Kit through JUUL’s e-commerce store between 4 April 2018 and 25 June 2018. The email invited individuals to participate in a 6-month online survey study about their use of combustible cigarettes, JUUL vapor products, and other e-cigarettes and vapor products. Invitations were sent to the email address associated with a customer’s age-verified verified account. Email invitations containing a web-link to the survey were scheduled to be sent to these individuals approximately 4 days after completing their online purchase of a JUUL Starter Kit so as to be received by the individual within 1–2 days after the scheduled delivery of their purchased product(s).

Second, individuals who purchased a JUUL Starter Kit in a retail store were invited to participate via 3″ ×  2.5″ cards that were manually inserted into the packaging of 500,000 JUUL Starter Kits, which were then distributed at random to approximately 10,000 licensed store retailers of JUUL vapor products across the USA. Starter Kits containing invitation cards were distributed across April 2018. Printed on each invitation card insert was the invitation text, the survey web address, and a unique six-digit alphanumeric code. Individuals who purchased a JUUL Starter Kit that contained an invitation card insert were invited to type the survey web address—survey.juul.com—into their web browser, and then, when prompted, type the six-digit code displayed on their invitation card insert. Entry of a valid code routed the individual to an Account Creation webpage, and then to the study Informed Consent Form. Each six-digit code was valid for one entry; attempts to re-use the code were blocked. Requiring the entry of a unique, one-time access code ensured that only individuals who had purchased a JUUL Starter Kit in a retail store could proceed to the Account Creation webpage, and requiring individuals to create a user account ensured that only one survey could be completed per account.

### Procedure

The first page of the survey displayed an Informed Consent Form (available upon request), which described the purpose of the survey, the names and contact details of the study investigators, information about who is eligible to take part and how survey data will be used, assurances of participant anonymity and confidentiality, and the source of funding for this study. Participants were informed that they were being invited to take part in six monthly online surveys about their use of combustible cigarettes, JUUL vapor products, and other e-cigarettes and vapor products. Individuals who satisfied eligibility criteria and gave informed consent to participate began the survey. Participants were routed to questions that were applicable to them on the basis of a response or combination of responses to a previous question or questions. The survey instrument was designed with the assumption that all respondents to a question would be asked the next question, unless there were specific instructions routing a subgroup of respondents to a different question. Participants answered survey questions at their own pace. If a participant did not complete the survey, all data provided up to the point of exit from the survey were not recorded.

The baseline survey took around 15–20 min to complete. Participants who completed the baseline survey received an automated email invitation to complete a follow-up survey every 30 ± 5 days for the next 6 months. An email invitation to participate in a follow-up survey was configured to be sent automatically to participants 25 days after the date of completion of the previous survey, with reminder emails sent 28 days and 31 days after the date of completion of the previous survey. Web-link access each follow-up survey expired 10 days after the first email invitation was sent. Participants received a USD$30 virtual Visa Reward Card by email for each survey they completed.

### Measures

#### Cigarette smoking in the past 30 days

The primary outcome measure in this study was past 30-day abstinence from smoking, which was determined at each assessment by a “No” response to the question, “In the past 30 days, have you smoked a cigarette, even one or two puffs?” Participants who indicated they have smoked a cigarette in the past 30 days were asked two further questions about their frequency of smoking in the past 30 days—“Do you now smoke cigarettes…” (every day; some days; not at all), and “On how many of the past 30 days did you smoke cigarettes?”^1^ (1–30 days)—and one question about their intensity of smoking in the past 30 days—“On those days that you did smoke, how many cigarettes did you usually smoke each day? A pack usually has 20 cigarettes in it”. Participants who did not provide valid answers to these four questions were excluded from the analytic sample.

#### Cigarette smoking history

Questions assessed the age at which participants first smoked a cigarette, first started smoking regularly, the number of months/years for which participants had been smoking cigarettes regularly, and the number of cigarettes participants had smoked in their lifetime.

#### Use of a JUUL vaporizer and JUULpod flavors in the past 30 days

Questions assessed the number of days in the past 30 days on which participants had used a JUUL vaporizer and the total number of JUULpods they had consumed in each of eight commercially available flavors (Virginia Tobacco, Mint, Mango, Crème, Fruit, Cucumber, Classic Tobacco, and Menthol) in the past 30 days. Participants were coded as a “primary user” of a specific flavor of JUULpod when they reported having consumed more pods in that flavor than in any other flavor. For example, a participant who reported having consumed ten Mango flavored JUULpods and five Mint flavored JUULpods in the past 30 days would be coded as a primary user of Mango flavored JUULpods.

Participants were coded as “past 30-day exclusive users to tobacco flavors” if they reported use of only Virginia Tobacco and/or Classic Tobacco in the past 30 days. Participants were coded as “past 30-day exclusive users to characterizing flavors” if they reported use of only Mint, Mango, Crème, Fruit, Cucumber, and/or Menthol in the past 30 days. Participants were coded as “past 30-day users of both tobacco and characterizing flavors” if they reported consumption of at least one pod in Virginia Tobacco or Classic Tobacco flavor and at least one pod in Mint, Mango, Crème, Fruit, Cucumber, or Menthol flavor.

#### Use of e-cigarettes other than a JUUL vaporizer in the past 30 days

Questions assessed participants’ frequency and intensity of use of e-cigarettes and vapor products other than JUUL vaporizer in the 30 days prior to the baseline survey. Participants who indicated they had used an e-cigarette other than a JUUL vaporizer in the past 30 days were asked about the characteristics of the e-cigarette they used most often in the past 30 days, including the brand of this e-cigarette, whether it was rechargeable and refillable, and what flavors and concentration of nicotine they used regularly in this e-cigarette/vapor product.

#### Reasons for purchasing and using a JUUL starter kit

At baseline, participants were asked to identify which, if any, of a list of health, social, financial, sensory, and convenience reasons were reasons why they first decided to purchase a JUUL Starter Kit.

#### Demographics

Questions assessed age, sex, census region, race-ethnicity, educational attainment, and annual household income.

### Data analysis

As this study is still collecting data, present analyses are restricted to data collected up to and including the 3-month follow-up survey assessment. Rates of past 30-day abstinence from smoking at the 3-month follow-up assessment are reported for the intention-to-treat (ITT) sample (*N* = 15,456) that completed the baseline survey assessment, stratified by place of first purchase of a JUUL Starter Kit (retail store purchasers *N* = 7823 vs. JUUL website purchasers *N* = 7633). In this analysis, at each follow-up assessment, participants with a missing response to the question “In the past 30 days, have you smoked a cigarette, even one or two puffs?” were recoded as “current smokers” under the worst-case scenario assumption that these participants had returned to baseline patterns of cigarette smoking.

Rates of past 30-day abstinence from smoking at the 3-month follow-up assessment are also reported for an efficacy subset comprising participants who provided smoking data at the 3-month follow-up assessment (*n* = 9272; 60.0% of the ITT sample), stratified by place of first purchase of a JUUL Starter Kit (retail store purchasers *n* = 4260 vs. JUUL website purchasers *n* = 5012). Rates of past 30-day point prevalence abstinence from smoking observed in the ITT sample and in the efficacy subset sample were therefore considered as lower and upper bound estimates of the rates of past 30-day point prevalence abstinence from smoking, respectively, at the 3-month follow-up assessment.

Factors associated with past 30-day abstinence from smoking at the 3-month assessment were examined through two logistic regression models, with each model conducted in two steps. In model 1 step 1, six demographic variables (age, sex, race/ethnicity, annual household income, education level, and US census region); four smoking history variables (age of first smoking, lifetime years of regular smoking, number of smoking days in the 30 days prior to the baseline assessment, number of cigarettes smoked per day in the 30 days prior to the baseline assessment); one e-cigarette use variable (current use of a secondary e-cigarette); and four JUUL use variables (place of first JUUL purchase, number of days of JUUL use in the past 30 days, primary JUULpod flavor used in the past 30 days, and having purchased a JUUL to help quit smoking) were entered as predictor variables. To assess the extent to which the effect of participants’ primary use of JUULpod flavors on past 30-day abstinence from smoking at the 3-month assessment varied by place at which participants purchased their first JUUL, an interaction term for “primary JUULpod flavor use”*“place of first JUUL purchase” was entered at step 2. Model 2 replicated model 1 with the variable “primary JUULpod flavor used in the past 30 days” replaced by the variable “JUULpod flavors used regularly in the past 30 days.” Odds ratios are reported unadjusted and adjusted for the effects of other variables in the model. Odds ratios in these regression models indicate the proportionate change in a participant’s odds of reporting past 30-day abstinence from smoking associated with the indicator on the categorical predictor variable. *P* values < 0.05 were considered statistically significant.

## Results

### Past 30-day point prevalence abstinence from smoking at 3-months assessment

**Fig. 1:**
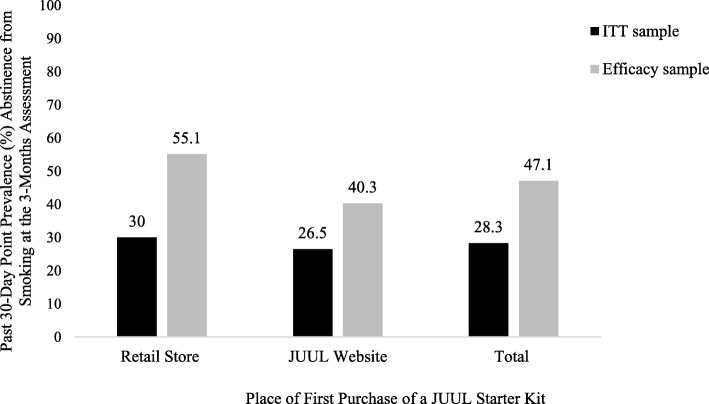
Past 30-day point prevalence abstinence from smoking at the 3-month assessment. Self-reported past 30-day point prevalence abstinence from cigarette smoking associated with using a JUUL vaporizer for three months, stratified by place of first purchase of a JUUL Starter Kit and sample type

In the ITT sample, overall past 30-day point prevalence abstinence from smoking at the 3-month assessment was 28.3% (*n* = 4367/15,456), with past 30-day point prevalence abstinence from smoking higher among retail purchasers (30.0%; *n* = 2346/7823) than among online purchasers (26.5%; *n* = 2021/7633). When the analysis was restricted to only those participants who completed the 3-month assessment, past 30-day point prevalence abstinence from smoking was 47.1% (*n* = 4367/9272), with past 30-day point prevalence abstinence from smoking higher among retail purchasers (55.1%; *n* = 2346/4260) than among online purchasers (40.3%; *n* = 2021/5012). We therefore estimate that, at the 3-month assessment, between 30.0% and 55.1% of new retail purchasers of a JUUL vaporizer and between 26.5% and 40.3% of new online purchasers of a JUUL vaporizer, all of whom were current smokers at the point of first purchase of a JUUL vaporizer, had not smoked a cigarette in the past 30 days (Fig. 1).

### Factors associated with past 30-day smoking abstinence at the 3-month assessment

Demographic, cigarette smoking, and e-cigarette use characteristics of participants who reported and did not report past 30-day smoking abstinence at the 3-month assessment are summarized in Table 1. In model 1 step 1, participants’ adjusted odds of reporting past 30-day abstinence from smoking significantly varied by four JUUL-related variables—primary JUULpod flavor used in the 30 days prior to the 3-month assessment; number of days of JUUL use in the 30 days prior to the 3-month assessment; place of first purchase of a JUUL Starter Kit; and whether or not participants first purchased a JUUL Starter Kit to help them quit smoking cigarettes—three smoking-related variables—number of smoking days in the 30 days prior to the baseline assessment, number of cigarettes smoked per day at the baseline assessment, and lifetime years of regular smoking—and one demographic variable—education level (Table 2).

**Table 1:** Demographic, smoking, and e-cigarette use characteristics of participants who completed the 3-month follow-up assessment (*n* = 9272; 60.0% of ITT sample), by smoking status at the 3-month follow-up assessment

Variable	Smoking status at 3-month follow-up assessment		
	Smoked in 30 days (*n* = 4905) *N* %	No smoking in past 30 days (*n* = 4367) *N* %	Total (*n* = 9272) *N* %
Demographic variables			
Sex			
Male	2716 (55.4)	2607 (59.7)	5323 (57.4)
Female	2120 (43.2)	1706 (39.1)	3826 (41.3)
Transgender	27 (0.6)	24 (0.5)	51 (0.6)
Missing	42 (0.9)	30 (0.7)	72 (0.8)
Age			
21–24	1339 (27.3)	1731 (39.6)	3070 (33.1)
25–34	1689 (34.4)	1413 (32.4)	3102 (33.5)
35–44	1003 (20.4)	621 (14.2)	1624 (17.5)
45–54	539 (11.0)	339 (7.8)	878 (9.5)
55–64	267 (5.4)	236 (5.4)	503 (5.4)
≥ 65	68 (1.4)	27 (0.6)	95 (1.0)
Race/Ethnicity			
Non-Hispanic, White	3439 (70.1)	2895 (66.3)	6334 (68.3)
Non-Hispanic, Black	149 (3.0)	143 (3.3)	292 (3.1)
Non-Hispanic, American Indian/Alaskan	61 (1.2)	54 (1.2)	115 (1.2)
Non-Hispanic, Asian, Hawaiian, or PI^a^	514 (10.5)	438 (10.0)	952 (10.3)
Non-Hispanic, two or more races	1 (0.0)	1 (0.0)	2 (0.0)
Hispanic^b^	390 (8.0)	427 (9.8)	817 (8.8)
Missing	351 (7.2)	409 (9.4)	760 (8.2)
Education			
Not HS graduate	139 (2.8)	115 (2.6)	254 (2.7)
GED	186 (3.8)	178 (4.1)	364 (3.9)
HS graduate	689 (14.0)	890 (20.4)	1579 (17.0)
Some college or associate’s degree	1901 (38.8)	1572 (36.0)	3473 (37.5)
Bachelor’s degree or higher	1778 (36.2)	1362 (31.2)	3140 (33.9)
Missing	212 (4.3)	250 (5.7)	462 (5.0)
Household income			
< $25,000	962 (19.6)	935 (21.4)	1897 (20.5)
$25,000 to $74,999	1979 (40.3)	1809 (41.4)	3788 (40.9)
≥ $75,000	1381 (28.2)	1126 (25.8)	2507 (27.0)
Missing	583 (11.9)	497 (11.4)	1080 (11.6)
U.S. census region			
Northeast	1109 (22.6)	908 (20.8)	2017 (21.8)
South	1785 (36.4)	1640 (37.6)	3425 (36.9)
Midwest	1159 (23.6)	1010 (23.1)	2169 (23.4)
West	813 (16.6)	782 (17.9)	1595 (17.2
Missing	39 (0.8)	27 (0.6)	66 (0.7)
Smoking and e-cigarette variables			
Age of first smoking			
≤ 11 years	191 (3.9)	126 (2.9)	317 (3.4)
12 to 14 years	1177 (24.0)	751 (17.2)	1928 (20.8)
15 to 17 years	1938 (39.5)	1552 (35.5)	3490 (37.6)
18 to 24 years	1497 (30.5)	1831 (41.9)	3328 (35.9)
≥ 25 years	87 (1.8)	94 (2.2)	181 (2.0)
Missing	15 (0.3)	13 (0.3)	28 (0.3)
Lifetime years of smoking			
≤ 1 year	315 (6.4)	513 (11.7)	828 (8.9)
1–5 years	1138 (23.2)	1485 (34.0)	2623 (28.3)
6–10 years	1071 (21.8)	870 (19.9)	1941 (20.9)
11–20 years	1265 (25.8)	784 (18.0)	2049 (22.1)
≥ 20 years	1029 (21.0)	601 (13.8)	1630 (17.6)
Missing	87 (1.8)	114 (2.6)	201 (2.2)
Number of smoking days in 30 days prior to baseline			
1–9 days	468 (9.5)	819 (18.8)	1287 (13.9)
10–19 days	456 (9.3)	686 (15.7)	1142 (12.3)
20–29 days	1031 (21.0)	1085 (24.8)	2116 (22.8)
30 days	2950 (60.1)	1777 (40.7)	4727 (51.0)
Cigarettes smoked per day at baseline			
1–9 cigarettes per day	2224 (45.3)	2626 (60.1)	4850 (52.3)
10–19 cigarettes per day	1609 (32.8)	1134 (26.0)	2743 (29.6)
≥ 20 cigarettes per day	1072 (21.9)	607 (13.9)	1679 (18.1)
Days of JUUL use in past 30 days at 3 months			
0 days	35 (0.7)	13 (0.3)	48 (0.5)
1–9 days	394 (8.0)	275 (6.3)	669 (7.2)
10–19 days	725 (14.8)	356 (8.2)	1081 (11.7)
20–29 days	1040 (21.2)	704 (16.1)	1744 (18.8)
30 days	2476 (50.5)	2710 (62.1)	5186 (55.9)
Missing	235 (4.8)	309 (7.1)	544 (5.9)
Current use of an e-cigarette other than JUUL			
Yes	568 (11.6)	407 (9.3)	975 (10.5)
No	4335 (88.4)	3958 (90.6)	8293 (89.4)
Missing	2 (0.0)	2 (0.0)	4 (0.0)
Place of first JUUL SK purchase			
Retail store	1914 (39.0)	2346 (53.7)	4260 (45.9)
JUUL website	2991 (61.0)	2021 (46.3)	5012 (54.1)
Bought JUUL SK ‘to help me quit smoking’			
Yes	4069 (83.0)	3670 (84.0)	7739 (83.5)
No	835 (17.0)	696 (15.9)	1531 (16.5)
Missing	1 (0.0)	1 (0.0)	2 (0.0)
Primary JUULpod flavor used in past 30 days (at 3 months)			
Virginia Tobacco	529 (10.8)	371 (8.5)	900 (9.7)
Mint	863 (17.6)	1008 (23.1)	1871 (20.2)
Mango	1107 (22.6)	1168 (26.7)	2275 (24.5)
Crème	258 (5.3)	191 (4.4)	449 (4.8)
Fruit	202 (4.1)	127 (2.9)	329 (3.5)
Cucumber	242 (4.9)	193 (4.4)	435 (4.7)
Classic Tobacco	138 (2.8)	40 (0.9)	178 (1.9)
Menthol	187 (3.8)	119 (2.7)	306 (3.3)
Equal use of 2+ flavors, no primary	1087 (22.2)	838 (19.2)	1925 (20.8)
Missing	292 (6.0)	312 (7.1)	604 (6.5)
JUULpod flavors used in past 30 days (at 3 months)			
Only used JUUL tobacco flavors^c^	472 (9.6)	277 (6.3)	749 (8.1)
Only used JUUL characterizing flavors^d^	3209 (65.4)	3194 (73.1)	6403 (69.1)
Used both tobacco and characterizing flavors	941 (19.2)	591 (13.5)	1532 (16.5)
Missing	283 (5.8)	305 (7.0)	588 (6.3)

*SK* JUUL starter kit (JUUL vaporizer plus four disposable pods); *3 M* 3 months follow-up assessment, *HS* high school, *GED* general educational development, *PI* Pacific Islander

^a^Includes Asian Indian, Chinese, Filipino, Japanese, Korean, Vietnamese, Guamanian, Chamorro, and Samoan

^b^Includes Mexican, Cuban, Puerto Rican and “other Hispanic” ethnicity

^c^JUUL tobacco flavors include “Virginia Tobacco” and “Classic Tobacco”

^d^JUUL characterizing flavors include “Mint,” “Mango,” “Crème,” “Fruit,” “Cucumber,” and “Menthol”

**Table 2:** Percent of participants reporting past 30-day point prevalence abstinence from smoking at the 3-month follow-up assessment and model information for two logistic regression analyses of factors associated with likelihood of reporting past 30-day abstinence from smoking at the 3-month follow-up assessment

Predictor variable	% P30A	Unadjusted	Model 1 adjusted		Model 2 adjusted	
			Step1	Step2	Step1	Step2
		Unadjusted OR (95% CI)	aOR (95% CI)	aOR (95% CI)	aOR (95% CI)	aOR (95% CI)
Sex						
Male	49.0	Ref.	Ref.	Ref.	Ref.	Ref.
Female	44.6	0.84 (0.77–0.91)***	0.93 (0.84–1.04)	0.93 (0.84–1.04)	0.92 (0.83–1.03)	0.93 (0.83–1.03)
Transgender	47.1	0.93 (0.53–1.61)	0.74 (0.38–1.45)	0.74 (0.38–1.43)	0.67 (0.35–1.30)	0.68 (0.35–1.31)
Age						
21–24	56.4	Ref.	Ref.	Ref.	Ref.	Ref.
25–34	45.6	0.65 (0.59–0.72)***	1.01 (0.87–1.18)	1.01 (0.87–1.18)	1.02 (0.87–1.19)	1.02 (0.87–1.19)
35–44	38.2	0.48 (0.42–0.54)***	0.93 (0.75–1.15)	0.93 (0.75–1.16)	0.93 (0.75–1.16)	0.93 (0.75–1.16)
45–54	38.6	0.49 (0.42–0.57)***	1.10 (0.84–1.46)	1.11 (0.84–1.46)	1.11 (0.84–1.46)	1.10 (0.84–1.46)
55–64	46.9	0.68 (0.57–0.83)***	0.91 (0.63–1.30)	0.90 (0.63–1.29)	0.91 (0.63–1.31)	0.91 (0.63–1.31)
≥ 65	28.4	0.31 (0.20–0.48)***	0.91 (0.47–1.75)	0.90 (0.47–1.74)	0.92 (0.48–1.76)	0.92 (0.48–1.76)
Race/Ethnicity						
Non-Hispanic, White	45.7	Ref.	Ref.	Ref.	Ref.	Ref.
Non-Hispanic, Black	49.0	1.14 (0.90–1.44)	1.01 (0.76–1.34)	1.01 (0.76–1.34)	0.99 (0.75–1.32)	0.99 (0.75–1.32)
Non-Hispanic, American Indian/Alaskan	47.0	1.05 (0.73–1.52)	1.33 (0.85–2.08)	1.33 (0.85–2.09)	1.28 (0.82–1.99)	1.27 (0.81–1.98)
Non-Hispanic, Asian, Hawaiian or PI^a^	46.0	1.01 (0.88–1.16)	0.91 (0.77–1.07)	0.90 (0.77–1.07)	0.91 (0.77–1.07)	0.91 (0.77–1.07)
Non-Hispanic, two or More Races	50.0	1.19 (0.07–19.00)	1.33 (0.08–22.13)	1.30 (0.08–21.72)	1.49 (0.09–24.76)	1.45 (0.09–23.85)
Hispanic^b^	52.3	1.30 (1.12–1.51)***	1.08 (0.91–1.29)	1.08 (0.91–1.29)	1.07 (0.90–1.28)	1.07 (0.90–1.28)
Education						
Not HS graduate	45.3	Ref.	Ref.	Ref.	Ref.	Ref.
GED	48.9	1.16 (0.84–1.60)	1.51 (1.02–2.22)*	1.51 (1.03–2.23)*	1.55 (1.05–2.28)*	1.55 (1.05–2.28)*
HS graduate	56.4	1.56 (1.20–2.04)***	1.34 (0.97–1.84)	1.34 (0.97–1.85)	1.37 (0.99–1.88)	1.37 (0.99–1.88)
Some college or associate’s degree	45.3	1.00 (0.77–1.29)	0.99 (0.72–1.34)	0.99 (0.73–1.36)	1.00 (0.74–1.36)	1.00 (0.74–1.36)
Bachelor’s degree or higher	43.4	0.93 (0.72–1.20)	0.88 (0.64–1.20)	0.89 (0.65–1.22)	0.90 (0.65–1.22)	0.89 (0.65–1.22)
Household income						
< $25,000	49.3	Ref.	Ref.	Ref.	Ref.	Ref.
$25,000 to $74,999	47.8	0.94 (0.84–1.05)	1.06 (0.93–1.21)	1.06 (0.93–1.22)	1.06 (0.93–1.21)	1.06 (0.93–1.21)
≥ $75,000	44.9	0.84 (0.74–0.95)***	1.12 (0.96–1.30)	1.12 (0.96–1.30)	1.13 (0.97–1.31)	1.13 (0.97–1.31)
U.S. census region						
Northeast	45.0	0.85 (0.75–0.97)*	0.97 (0.82–1.15)	0.97 (0.82–1.14)	0.98 (0.83–1.15)	0.98 (0.83–1.15)
South	47.9	0.96 (0.85–1.08)	1.10 (0.94–1.28)	1.09 (0.94–1.27)	1.11 (0.95–1.29)	1.11 (0.95–1.29)
Midwest	46.6	0.91 (0.80–1.03)	0.95 (0.80–1.12)	0.94 (0.80–1.11)	0.95 (0.81–1.12)	0.95(0.81–1.12)
West	49.0	Ref.	Ref.	Ref.	Ref.	Ref.
Age of first smoking						
≤ 11 years	39.7	Ref.	Ref.	Ref.	Ref.	Ref.
12 to 14 years	39.0	0.97 (0.76–1.23)	0.87 (0.65–1.17)	0.88 (0.65–1.17)	0.87 (0.65–1.17)	0.87 (0.65–1.16)
15 to 17 years	44.5	1.21 (0.96–1.54)	0.93 (0.70–1.24)	0.93 (0.70–1.24)	0.92 (0.69–1.23)	0.92 (0.69–1.22)
18 to 24 years	55.0	1.85 (1.47–2.35)***	1.15 (0.86–1.54)	1.15 (0.86–1.54)	1.14 (0.85–1.53)	1.14 (0.85–1.52)
≥ 25 years	51.9	1.64 (1.13–2.37)**	1.34 (0.82–2.19)	1.35 (0.83–2.20)	1.32 (0.81–2.15)	1.31 (0.81–2.14)
Lifetime years of smoking						
≤ 1 year	62.0	2.79 (1.22–1.59)***	1.78 (1.29–2.44)***	1.78 (1.30–2.45)***	1.80 (1.31–2.48)***	1.80 (1.31–2.48)***
1–5 years	56.6	2.23 (1.97–2.54)***	1.57 (1.20–2.05)**	1.57 (1.20–2.05)**	1.58 (1.21–2.07)**	1.59 (1.21–2.07)**
6–10 years	44.8	1.39 (2.35–3.31)***	1.26 (0.99–1.61)	1.27 (0.99–1.62)	1.28 (1.00–1.63)*	1.28 (1.00–1.64)*
11–20 years	38.3	1.06(0.93–1.21)	1.14 (0.92–1.40)	1.14 (0.92–1.40)	1.14 (0.93–1.41)	1.15 (0.93–1.41)
≥ 20 years	36.9	Ref.	Ref.	Ref.	Ref.	Ref.
Number of smoking days in 30 days prior to baseline						
1–9 days	63.6	2.91 (2.56–3.30)***	2.87 (2.40–3.43)***	2.88 (2.41–3.44)***	2.86 (2.40–3.42)***	2.86 (2.40–3.42)***
10–19 days	60.1	2.50 (2.19–2.85)***	2.28 (1.91–2.71)***	2.28 (1.92–2.72)***	2.26 (1.90–2.69)***	2.26 (1.90–2.69)***
20–29 days	51.3	1.75 (1.58–1.94)***	1.55 (1.36–1.77)***	1.56 (1.36–1.78)***	1.55 (1.36–1.78)***	1.55 (1.36–1.77)***
30 days	37.6	Ref.	Ref.	Ref.	Ref.	Ref.
Cigarettes smoked per day at baseline						
1–9 cigarettes per day	54.1	Ref.	Ref.	Ref.	Ref.	Ref.
10–19 cigarettes per day	41.3	0.60 (0.54–0.66)***	0.91 (0.80–1.03)	0.91 (0.80–1.04)	0.91 (0.80–1.04)	0.91 (0.80–1.04)
≥ 20 cigarettes per day	36.2	0.48 (0.43–0.54)***	0.84 (0.71–0.99)*	0.84 (0.71–0.99)*	0.84 (0.71–0.99)*	0.84 (0.71–0.99)*
Number of days of JUUL use in past 30 days (at 3 months)						
0 days	27.1	0.34 (0.18–0.64)	–	–	–	–
1–9 days	41.1	0.64 (0.54–0.75)	0.56 (0.45–0.68)***	0.55 (0.45–0.68)***	0.53 (0.43–0.65)***	0.53 (0.43–0.65)***
10–19 days	32.9	0.45 (0.39–0.52)	0.38 (0.32–0.45)***	0.38 (0.32–0.45)***	0.36 (0.31–0.43)***	0.36 (0.31–0.43)***
20–29 days	40.4	0.62 (0.55–0.69)	0.51 (0.45–0.58)***	0.51 (0.44–0.58)***	0.50 (0.44–0.57)***	0.50 (0.44–0.57)
30 days	52.3	Ref.	Ref.	Ref.	Ref.	Ref.
Current use of an e-cigarette other than JUUL						
Yes	41.7	0.79 (0.69–0.90)***	0.97 (0.82–1.15)	0.97 (0.81–1.15)	0.97 (0.82–1.16)	0.98 (0.82–1.16)
No	47.7	Ref.	Ref.	Ref.	Ref.	Ref.
Place of first JUUL SK purchase						
Retail store	55.1	1.81 (1.67–1.97)***	1.37 (1.22–1.53)***	1.39 (0.99–1.94)	1.38 (1.23–1.54)***	1.54 (1.04–2.26)*
JUUL website	40.3	Ref.	Ref.	Ref.	Ref.	Ref.
Bought JUUL SK “to help me quit smoking”						
Yes	47.4	1.08 (0.97–1.21)	1.34 (1.16–1.55)***	1.35 (1.17–1.55)***	1.36 (1.18–1.57)***	1.36 (1.18–1.57)***
No	45.5	Ref.	Ref.	Ref.	Ref.	Ref.
Primary JUULpod flavor used in past 30 days (at 3 months)						
Virginia Tobacco	41.2	Ref.	Ref.	Ref.	Ref.	Ref.
Mint	53.9	1.67 (1.42–1.96)***	1.37 (1.13–1.66)**	1.42 (1.11–1.81)**	NI	NI
Mango	51.3	1.50 (1.29–1.76)***	1.26 (1.05–1.52)*	1.27 (1.01–1.61)*	NI	NI
Crème	42.5	1.06 (0.84–1.33)	1.13 (0.86–1.48)	1.16 (0.83–1.62)	NI	NI
Fruit	38.6	0.90 (0.69–1.16)	0.94 (0.69–1.29)	0.96 (0.63–1.46)	NI	NI
Cucumber	44.4	1.14(0.90–1.43)	0.88 (0.67–1.16)	0.85 (0.60–1.21)	NI	NI
Classic Tobacco	22.5	0.41 (0.28–0.60)***	0.54 (0.35–0.84)**	0.51 (0.30–0.86)*	NI	NI
Menthol	38.9	0.91 (0.70–1.18)	0.90 (0.66–1.24)	1.12 (0.76–1.66)	NI	NI
Equal use of 2+ flavors, no primary	43.5	1.10 (0.94–1.29)	0.99 (0.82–1.20)	0.93 (0.73–1.19)	NI	NI
JUULpod flavors used in the past 30 days (at 3 months)						
Only used JUUL tobacco flavors^c^	37.0	Ref.	NI	NI	Ref.	Ref.
Only used JUUL characterizing flavors^d^	49.9	1.70 (1.45–1.98)***	NI	NI	1.30 (1.07–1.57)**	1.33 (1.05–1.67)*
Used flavors from both tobacco and categories	38.6	1.07 (0.89–1.28)	NI	NI	0.88 (0.71–1.09)	0.97 (0.74–1.27)
Interaction term: primary JUULpod flavor used in past 30 days (at 3 months)^c^ Place of first JUUL SK purchase						
Virginia Tobacco^c^ Retail	–	–	–	Ref.		
Mint^c^ Retail	–	–	–	0.92 (0.62–1.37)	NI	NI
Mango^c^ Retail	–	–	–	0.98 (0.67–1.44)	NI	NI
Crème^c^ Retail	–	–	–	0.93 (0.53–1.63)	NI	NI
Fruit^c^ Retail	–	–	–	0.96 (0.52–1.80)	NI	NI
Cucumber^c^ Retail	–	–	–	1.08 (0.61–1.89)	NI	NI
Classic Tobacco^c^ Retail	–	–	–	1.25 (0.47–3.35)	NI	NI
Menthol^c^ Retail	–	–	–	0.55 (0.28–1.06)	NI	NI
Equal use of 2+ flavors, no primary^c^ Retail	–	–	–	1.14 (0.77–1.69)	NI	NI
Interaction term: JUULpod flavors used in the past 30 days (at 3 months)^c^ place of first JUUL purchase						
Only JUUL tobacco flavors^c^ Retail	–	–	–	NI	–	Ref.
Only JUUL characterizing flavors^c^ Retail	–	–	–	NI	–	0.92 (0.61–1.37)
Both flavor categories and tobacco^c^ Retail	–	–	–	NI	–	0.77 (0.49–1.21)

Model 1: *N* = 6968, χ^2^ = 836.329, df = 49, *p <* 0.001

Model 2: *N* = 6979, χ^2^ = 823.798, df = 43, *p <* 0.001

*P30A* past 30-day abstinence from smoking at the 3-month assessment, *SK* JUUL starter kit (JUUL vaporizer plus four disposable pods), *3 M* 3-month assessment, *aOR* adjusted odds ratio, *HS* high school, *CPD* cigarettes smoked per day, *PI* Pacific Islander, *NI* not included in the logistic regression model

Unadjusted ORs were estimated using only the relevant variable as the predictor variable

***p < 0.001; **p < 0.010; *p < 0.050

^a^Includes Asian Indian, Chinese, Filipino, Japanese, Korean, Vietnamese, Guamanian, Chamorro, and Samoan

^b^Includes Mexican, Cuban, Puerto Rican and “other Hispanic” ethnicity

cJUUL tobacco flavors include “Virginia Tobacco” and “Classic Tobacco”

^d^JUUL characterizing flavors include “Mint,” “Mango,” “Crème,” “Fruit,” “Cucumber,” and “Menthol”

#### Model 1

##### JUUL-related predictors

Compared to those who primarily used Virginia Tobacco flavored JUULpods in the 30 days prior to the 3-month assessment, those who primarily used Mint flavored JUULpods (aOR = 1.37; 1.13, 1.66) or Mango flavored JUULpods (aOR = 1.26; 1.05, 1.52) were 37% and 26% more likely, respectively, to have not smoked a cigarette in the 30 days prior to the 3-month assessment. Mint and Mango were the most common primary flavors, with primary users of Mint and Mango flavored JUULpods in the 30 days prior to the 3-month assessment together accounting for 49.8% of all participants who had not smoked a cigarette in the 30 days prior to the 3-month assessment, and 44.7% of all participants who completed the 3-month assessment. Compared to those who primarily used Virginia Tobacco flavored JUULpods, those who primarily used Classic Tobacco flavored JUULpods (aOR = 0.54; 0.35, 0.84) were 1.85 times less likely to have not smoked a cigarette in the 30 days prior to the 3-month assessment. Compared to those who primarily used Virginia Tobacco flavored JUULpods, odds for reporting past 30-day smoking abstinence at the 3-month assessment were not significantly different among those who primarily used Crème, Fruit, Cucumber, or Menthol flavored JUULpods, or among those who did not have a primary flavor in the 30 days prior to the 3-month assessment. The interaction term entered at step 2 was non-significant, indicating that the association between primary JUULpod flavor used in the 30 days prior to the 3-month assessment and past 30-day smoking abstinence at the 3-month assessment was not significantly moderated by the place at which participants purchased their first JUUL vaporizer.

Compared to those who purchased their first JUUL vaporizer through the e-commerce store on JUUL’s website, those who purchased their first JUUL vaporizer in a retail store were 37% more likely to have not smoked a cigarette in the 30 days prior to the 3-month assessment (aOR = 1.37; 1.22, 1.53). Compared to those who used a JUUL vaporizer on all 30 of the 30 days prior to the 3-month assessment, those who used a JUUL vaporizer on 20–29 days (aOR = 0.51; 0.45, 0.58), 10–19 days (aOR = 0.38; 0.32, 0.45), and 1–9 days (aOR = 0.56; 0.45, 0.68) in the past 30 days were 1.96 times, 2.63 times, and 1.79 times less likely, respectively, to have not smoked a cigarette in the 30 days prior to the 3-month assessment. Compared to those who did not purchase their first JUUL vaporizer in order to help them to quit smoking cigarettes completely, those who did purchase their first JUUL vaporizer in order to help them to quit smoking cigarettes completely were 34% more likely to have not smoked a cigarette in the 30 days prior to the 3-month assessment (aOR = 1.34; 1.16, 1.55).

##### Smoking-related predictors

Smoking heaviness, frequency, and duration at the point of first purchase of a JUUL Starter Kit were all negatively associated with participants’ odds of reporting past 30-smoking abstinence at the 3-month assessment. Compared to those who had smoked cigarettes on all 30 of the 30 days prior to the baseline assessment, those who had smoked cigarettes on 20–29 days (aOR = 1.55; 1.36, 1.77), 10–19 days (aOR = 2.28; 1.91, 2.71), and 1–9 days (aOR = 2.87; 2.40, 3.43) of the 30 days prior to the baseline assessment were approximately 1.6 times, 2.3 times, and 2.9 times more likely to have not smoked a cigarette in the 30 days prior to the 3-month assessment. Compared to those who were smoking 1–9 cigarettes per day at the baseline assessment, those who were smoking 20 or more cigarettes per day at the baseline assessment were 19% less likely to have not smoked a cigarette in the 30 days prior to the 3-month assessment (aOR = 0.84; 0.71, 0.99). Compared to those had smoked regularly for 20 or more years in their lifetime at the baseline assessment, those who had smoked regularly for 0–12 months (aOR = 1.78; 1.29, 2.44) and 1–5 years (aOR = 1.57; 1.20, 2.05) were 78% and 57% more likely, respectively, to have not smoked a cigarette in the 30 days prior to the 3-month assessment.

##### Demographic predictors

Compared to those who had not graduated high school, those with a General Education Diploma (GED) were 51% more likely to have not smoked a cigarette in the 30 days prior to the 3-month assessment (aOR = 1.51; 1.02, 2.22).

#### Model 2

All eight variables that emerged as significant predictors of past 30-day smoking abstinence at the 3-months assessment in model 1 remained significant in model 2, with no non-significant predictors in model 1 becoming significant in model 2. The added variable—*JUULpod flavors used in the 30 days prior to the 3*-*month assessment*—was significantly associated with participants’ odds of reporting past 30-day abstinence from smoking at the 3-month assessment. Compared to those who exclusively used JUULpods in tobacco flavors in the 30 days prior to the 3-month assessment, those who exclusively used JUULpods in characterizing flavors were 30% more likely to have not smoked a cigarette in the 30 days prior to the 3-month assessment (aOR = 1.30; 1.07, 1.57). Compared to those who exclusively used JUULpods in tobacco flavors in the 30 days prior to the 3-month assessment, those who had used JUULpods in both tobacco and characterizing flavors in the past 30 days were non-significantly less likely to have not smoked a cigarette in the 30 days prior to the 3-month assessment (aOR = 0.88; 0.71, 1.09). Finally, the interaction term entered at step 2 was non-significant, indicating that the association between past 30-day use of JUULpod flavor categories at the 3-month assessment and past 30-day smoking abstinence at the 3-month assessment was not significantly moderated by the place at which participants purchased their first JUUL vaporizer.

## Discussion

At least 28.3% of a large non-probabilistic sample of US adult established current smokers had not smoked any cigarettes for at least 30 days when assessed 3 months after purchasing their first JUUL vaporizer from a retail store or e-commerce website. Of those who responded to the 3-month assessment, 47.1% reported having not smoked any cigarettes for at least the past 30 days. Eight variables were significant in predicting smokers’ likelihood of reporting past 30-day abstinence from smoking after using a JUUL vaporizer for 3 months. Daily use of a JUUL vaporizer in the past month, primary use of Mint or Mango flavored JUULpods, exclusive use of JUULpods in characterizing flavors, purchasing one’s first JUUL Starter Kit in a retail store, and purchasing one’s first JUUL vaporizer to help to quit smoking completely were all associated with significantly higher adjusted odds of not having smoked any cigarettes in the 30 days prior to the 3-month assessment. Smoking regularly for more than 20 years, smoking more cigarettes per day and smoking on more days in the 30 days before purchasing one’s first JUUL Starter Kit, and primary use of Classic Tobacco flavored JUULpods (versus Virginia Tobacco flavored pods) were all associated with significantly lower adjusted odds of not having smoked any cigarettes in the 30 days prior to the 3-month assessment.

JUULpods in non-tobacco flavors, particularly Mint and Mango, appeared to play an important role in helping smokers to quit within the first 3 months of using a JUUL vaporizer. In addition to being the most commonly used flavors in the JUULpod flavor range, smokers who *primarily* vaped JUULpods flavored to taste like Mint or Mango in the 30 days prior to the 3-month assessment were 37% and 26% more likely, respectively, to have not smoked any cigarettes in the 30 days prior to the 3-month assessment (compared to primary users of Virginia Tobacco flavored JUULpods). Likewise, smokers who had *exclusively* used JUULpods in characterizing flavors—Mint, Menthol, Mango, Cucumber, Fruit, and/or Crème—in the 30 days prior to the 3-month assessment were 30% more likely to have not smoked any cigarettes in the 30 days prior to the 3-month assessment (compared to exclusive users of JUULpods in tobacco flavors).

Purchasing one’s first JUUL Starter Kit in a retail store also appeared to play an important role in the success of smokers’ quit attempts. Compared to smokers who purchased their first JUUL Starter Kit through the e-commerce store at JUUL’s website, smokers who purchased their first JUUL Starter Kit in a retail store were 37% more likely to have not smoked any cigarettes in the 30 days prior to the 3-month assessment. Additionally, a non-significant interaction term indicated that the beneficial effect of primary use of Mint or Mango pods (versus Virginia Tobacco pods) was statistically equivalent for individuals who purchased their first JUUL Starter Kit in a retail store and for individuals who purchased their first JUUL Starter Kit through the e-commerce store on JUUL’s website.

Together with evidence that use of non-tobacco flavors (versus tobacco flavors) was more strongly associated with 3-month quit outcomes, the finding that smokers who purchased their first JUUL Starter Kit and e-liquid pods in a retail store are more likely to have quit smoking 3 months later is significant in the light of an announcement from JUUL Labs Inc. on 13 November 2018. This announcement stated that, in response to concern expressed by the FDA about the role of non-tobacco flavors in increasing the appeal of vaping to youth, JUUL Labs Inc. has stopped selling its Mango, Crème, Fruit, and Cucumber flavored pods to the over 90,000 retail stores in the USA that currently sell JUUL’s flavored pods, including convenience stores and specialty vape stores. This voluntary action by JUUL Labs Inc. to suspend retail sales of flavored products preceded an announcement by FDA on 15 November 2018 of an intention to revisit its discretionary extension of the premarket application compliance date to August 2022 for newly regulated non-combustible tobacco products that are flavored, including all flavors other than tobacco, mint, and menthol. The changes being sought by FDA Commissioner Gottlieb would require all flavored ENDS products (other than tobacco, mint, and menthol flavors or non-flavored products) sold in age-restricted, in-person locations, and, if sold online, under heightened practices for age verification.

FDA’s proposal to ban the retail sale of flavored ENDS products is an effort to strike a careful balance between maintaining adult smokers’ access to potentially less harmful sources of nicotine through ENDS for adults who want to transition away from combustible cigarettes, and reducing youth appeal and access to ENDS products. This policy will mean that, in the near future, adult smokers aged 21 years and older in the USA who want to purchase JUULpods flavored to taste like Mango, Crème, Fruit, and Cucumber will only be able to do so (legally) through the e-commerce store at JUUL’s website. Adult smokers will still be able to purchase Mint flavored pods in retail stores, which present evidence suggests is both the most popular flavor and the flavor most strongly associated with past 30-day smoking abstinence at the 3-month assessment. Adult smokers will only be able to purchase JUULpods in four flavors in retail stores, three of which—Virginia Tobacco, Classic Tobacco, and Menthol—are considerably less popular than Mint and Mango and associated with significantly lower 3-month quit rates compared to Mint and Mango. In light of data from the present study then, the restrictions imposed by this policy will mean adults will be less able to purchase the flavors of JUULpods that are the most preferred and most strongly associated with short-term quitting success, from the points of purchase that are more strongly associated with short-term quitting success. It will therefore be vitally important to measure the impact of suspending retail sales of flavored JUULpods on rates of use of JUULpods by both adult smokers and youth, and in turn, on associated rates of smoking cessation and initiation. In particular, there is a need for population surveillance systems through which researchers can compare the strength of the prospective association between adults’ use of flavored ENDS products and smoking cessation at the population level, and the prospective association between youths’ use of flavored ENDS products and smoking initiation at the population level.

It is possible that banning retail sales of flavored ENDS products could have little impact on adult smoking quit rates if smokers simply migrate to a retail-available second or third flavor choice that they also find to be a satisfying alternative to cigarettes. Present evidence of the stronger association between primary use of Mint and Mango JUULpods should not be interpreted as evidence that the retail availability of JUULpods in these flavors is *essential* to increasing rates of smoking cessation. It is possible that, when unable to purchase Mint and Mango pods in retail stores, a proportion of smokers who would prefer to purchase these flavors will switch to using JUULpods in the tobacco and mint/menthol flavors that will continue to be available, rather than discontinuing their use of a JUUL vaporizer altogether. The uncontrolled nature of our study design means we cannot know what proportion of those quitters who were retail purchasers and primary users of Mango flavored JUULpods would have used JUULpods in tobacco or mint/menthol flavors and quit smoking even if Mango flavored JUULpods had been unavailable for purchase in retail stores during this study.

It is perhaps more plausible, however, to expect the loss of retail store access to Mango flavored JUULpods—the second most popular flavor and a flavor associated with a significantly higher 3-month quit rate than flavors that will still be available for purchase in retail stores (Virginia Tobacco, Classic Tobacco, and Menthol)—will result in fewer adult smokers achieving smoking abstinence in the first 3 months of using a JUUL vaporizer, and may result in an increased drop-off in the number of primary retail-Mango pod users who had quit smoking at the 3-month assessment remaining quit at the 6-month assessment of this study. This latter hypothesis will be addressed by data from the 6-month assessment of this study when available.

A third and much less likely possibility is that the proportion of smokers who achieved smoking abstinence in the third month of using a JUUL vaporizer in this study would have been higher had Mango flavored JUULpods not been available to purchase in retail stores during the period of this study. Given evidence that Mint and Mango flavored JUULpods are more strongly preferred by adult smokers to JUULpods in tobacco flavors, and significantly more strongly associated with smoking cessation within the first 3 months of using a JUUL vaporizer, regulatory actions that FDA may take to prevent youth access to flavored ENDS products that also have the effect of preventing or reducing adult smokers’ access to JUULpods in these non-tobacco flavors risk losing a substantial number of successful smoking quit attempts that are solely or sufficiently attributable to use of JUULpods in these non-tobacco flavors. In this sense, the present study may provide useful reference information for studies of a similar nature conducted post-implementation of any policies that affect adult smokers’ access to flavored ENDS products.

Smokers who used a JUUL vaporizer more frequently in the 30 days prior to the 3-month assessment were also significantly more likely to have not smoked a cigarette in those 30 days. This is consistent with findings from nationally representative surveys [16, 17]. For example, data from the 2014 and 2015 US National Health Interview Survey (NHIS) showed that over half (52%) of daily e-cigarette users had quit smoking in the last 5 years. Daily e-cigarette users were 3.15 times more likely to have quit smoking compared to those who have never used an e-cigarette (28.2%). Those who used e-cigarettes on only some days were least likely to have quit (12.1%). The observation of similarly strong associations between daily use of a JUUL vaporizer and past 30-day cigarette abstinence outcomes in this study reinforce the notion that adults who are using a JUUL vaporizer to help them to quit smoking should be encouraged to use their JUUL vaporizer as needed each day.

Adult smokers in this study were, at the point of their first purchase of a JUUL Starter Kit, more likely to be males aged 21–34, daily smokers of 1–9 cigarettes, and smoking cigarettes regularly for less than 10 years. Consistent with the literature on the negative association between severity of cigarette dependence and odds for quitting smoking, participants’ likelihood of completely switching from combustible cigarettes to a JUUL vaporizer after 3 months decreased as their frequency, heaviness, and duration of cigarette smoking at baseline increased. These data have several important implications for the current and potential impact of JUUL vapor products on smoking cessation and smoking-related disease in the US adult population.

First, at present, new JUUL users are more likely to be lighter smokers who have been smoking for fewer years of their lifetime, and present data indicate that odds for quitting smoking within 3 months of initiating use of a JUUL vaporizer are highest among these smoker groups. By appealing more to younger, lighter, shorter-term smokers who, by virtue of their reduced heaviness and duration of exposure to cigarette smoke are likely to be presently experiencing fewer health problems related to cigarette smoking, and by being more effective in helping these smokers to quit within 3 months, the use of a JUUL vaporizer for even a short period of time may be highly effective in diverting younger, less dependent smokers away from smoking before they begin to experience serious smoking-related health problems and become increasingly dependent on cigarettes. However, given that lighter smokers may have been comparably likely to try and succeed in quitting smoking with non-pharmacological interventions, and given that long-term inhalation of vapor from a JUUL vaporizer is unlikely to be without some health risks, the potential benefits of switching from light smoking to regular use of JUUL vaporizer need to be weighed against the potential risks that could be incurred through prolonged exposure to daily, high doses of nicotine and varying levels of other harmful and potentially harmful constituents of vapor if these switchers were to go on to become long-term or lifelong JUUL users.

Second, there is a need to understand the extent to which the lower proportions of older, heavier, longer-term, and female cigarette smokers who opted into this study are reflected in the whole population of JUUL users, and if so, why these smoking sub-groups, who are at greater risk for developing smoking-related diseases, are less likely to use JUUL vapor products. There is also a clear need to better understand why, beyond lifetime cigarette exposure, smokers who are at the greatest risk of developing smoking-related diseases—those smoking more cigarettes per day, smoking more days in the month, and have smoked for more years in their lifetime at the point of first purchase of a JUUL Starter Kit—have the greatest difficulty in completely substituting a JUUL vaporizer for combustible cigarettes within 3 months. Specifically, research should seek to understand the extent to which these smokers’ lower likelihood of using a JUUL vaporizer and lower likelihood of completely switching to a JUUL vaporizer could be addressed by innovating the look, feel, taste, nicotine delivery, and satisfaction of existing JUUL products to be increasingly socially acceptable and pharmacologically appealing to heavier, more frequent, and longer-term cigarette smokers, and by increasing marketing of JUUL vapor products toward these smoking sub-groups. It should be acknowledged, however, that, for many smokers, there may be no level of innovation of an e-cigarette that will replicate or compete with the satisfaction of smoking a cigarette. Assisting these individuals to quit smoking sooner may require provision of adjunctive behavioral support, concurrent use of other products and methods that are empirically supported for smoking cessation, and/or education and practical skills training on how to use JUUL vapor products to maximize their chances of quitting smoking.

A final important finding of this study was that, though the quit rate at 3 months was significantly higher among smokers who purchased their first JUUL Starter Kit to help them to quit smoking cigarettes, the unadjusted quit rate (45.5%) among the 17% of smokers who did not buy their first JUUL Starter Kit to help to quit smoking was also high. This finding suggests that many smokers who had initiated use of a JUUL vaporizer with an intention to dual use cigarettes and the JUUL vaporizer ultimately came to prefer exclusive use of a JUUL vaporizer to dual use of both products. That a high proportion of smokers without an initial intention to quit smoking ultimately did quit smoking is encouraging given that many smokers tend to initiate use of e-cigarettes primarily out of curiosity about the taste and effects of vaping, and with skepticism about the extent to which vaping can replace, let alone compete with, smoking cigarettes. Smokers may perceive a need to use a JUUL vaporizer, or indeed any e-cigarette, for at least several weeks or months to be assured that e-cigarettes can meet their wants and needs that have long been well served by cigarettes, and so increase initially low levels of interest, motivation, and self-efficacy for switching to levels necessary for them to begin to contemplate and then attempt to switch completely away from cigarettes. Future research that identifies the typical duration of use a JUUL vaporizer at which smokers whom had an initial intention to use a JUUL vaporizer in addition to cigarettes come to formalize and pursue a long-term goal of using a JUUL vaporizer in place of cigarettes could provide crucial insights into the early subjective experiences (e.g., sensorial, hedonic, physical, emotional) of using a JUUL vaporizer that prompt smokers to re-evaluate their motivation and perceived need to continue smoking cigarettes.

The conclusions of this study are limited in several ways. First, the sample is not representative of the US adult population of smokers or e-cigarette users, nor was the study designed or intended to estimate the prevalence or frequency of use of JUUL vapor products among the e-cigarette-using population of US adults. The study aimed to elicit data on individual characteristics and patterns of cigarette smoking and use of JUUL vapor products that are prospectively associated with increased and reduced odds of quitting smoking in a large, self-selecting sample of adult established current smokers who had very recently started to use a JUUL vaporizer and who agreed to participate in each survey in exchange for US$30. The recruitment methods were therefore biased toward outlets where new JUUL users would be found, and so the study conclusions do not represent the individual user characteristics, patterns of product use, or smoking outcomes associated with any other ENDS product, and may also not represent new JUUL purchasers who declined the invitation to participate in this study.

By including only those who were adult established current smokers at the time of their first purchase of a JUUL Starter Kit, this study additionally does not yield data on the proportion of all new JUUL purchasers who are adults (versus adolescents) or current smokers (versus former smokers and never smokers). In turn, this study yields no data about the rate of smoking initiation and smoking relapse among those who were not actively smoking or had never smoked a cigarette, respectively, when they purchased their first JUUL Starter Kit. Estimating these rates are essential for modeling the impact of using JUUL vapor products on the health of the whole US population, the majority of which are non-users of tobacco products.

We must also stress that the short-term self-reported smoking outcome data reported here should not be taken as evidence that using a JUUL vaporizer can be effective for helping smokers to quit in the long-term. To our knowledge, no data have been published that describe the extent to which use of a JUUL vaporizer is associated with long-term abstinence from cigarette smoking, or the extent to which short-term changes in smoking behavior associated with JUUL use are predictive of longer-term, clinically significant changes in smoking behavior, or smoking-related health outcomes. The extent to which study participants who reported positive short-term smoking behavior change after 3 months of using a JUUL vaporizer ultimately relapse to baseline smoking patterns or sustain early positive changes up to and including 6 months of JUUL use will be examined when the data from the 6-months assessment of this study become available.

Finally, we stress that the data presented here on the rates of quitting smoking associated with different patterns of JUUL use for 3 months do not permit conclusions about the potential impact of JUUL use on the current or future health status of study participants. No data have been presented that would permit the conclusion that adults who switched completely from smoking cigarettes to using a JUUL are likely to have increased or reduced their exposure to harmful and potentially harmful toxicants, or their risk for developing serious health problems. The present study collected no data that could adequately characterize the health impact of switching from smoking cigarettes to using a JUUL vaporizer. Studies that characterize the risk/safety profile of JUUL vapor products relative to combustible cigarettes, other ENDS products, and FDA-approved smoking cessation products and medications, and which characterize the patterns of use of JUUL vapor products that increase and decrease risks to users’ health, are urgently needed.

## Conclusions

Between 28.3% and 47.1% of adult new users of the JUUL vaporizer, who were established daily or non-daily smokers when they began using a JUUL vaporizer, had not smoked any cigarettes in the third month of using a JUUL vaporizer. Those who used a JUUL vaporizer every day, purchased their first JUUL Starter Kit in a retail store, primarily used JUULpods flavored to taste like Mint or Mango, and exclusively used JUULpods in characterizing flavors were significantly more likely to have not smoked any cigarettes in the third month of using a JUUL vaporizer. Given this evidence of the importance of JUULpods containing characterizing flavors—and their availability in retail stores—to smokers’ likelihood of quitting smoking in the short-term, the impact of a pending suspension of retail sales of flavored JUULpods on adults’ likelihood of quitting smoking should be closely assessed. Present data may be used as reference information for assessing the impact of policies that restrict the accessibility of flavored JUUL vapor products on the rate of smoking cessation among adults who attempt to switch completely to JUUL vapor products. As part of a broader collection of data on the human health impact of JUUL vapor products, these data can also assist the FDA Center for Tobacco Products to determine whether issuing a marketing authorization order for JUUL vapor products would be appropriate for the protection of the public health under section 910 of the FD&C Act (21 U.S.C. 387j).

## Footnotes

1 This question was only asked to those who reported smoking on “some days.” A value of “30” was imputed for those who reported having smoked ‘every day’ in the past 30 days.
